# Crystal structure of (15,20-bis­(2,3,4,5,6-penta­fluoro­phen­yl)-5,10-{(pyridine-3,5-di­yl)bis­[(sulfane­diyl­methyl­ene)[1,1′-biphen­yl]-4′,2-di­yl]}porph­yrin­ato)nickel(II) di­chloro­methane *x*-solvate (*x* > 1/2) showing a rare CN5 coordination

**DOI:** 10.1107/S2056989019009836

**Published:** 2019-07-12

**Authors:** Florian Gutzeit, Christian Näther, Rainer Herges

**Affiliations:** a Otto-Diels-Institut für Organische Chemie, Christian-Albrechts-Universität Kiel, Otto-Hahn-Platz 4, D-24098 Kiel, Germany; b Institut für Anorganische Chemie, Christian-Albrechts-Universität Kiel, Max-Eyth Str. 2, D-24118 Kiel, Germany

**Keywords:** crystal structure, nickel porphyrin, square-pyramidal coordination, hydrogen bonding

## Abstract

The crystal structure of the title compound, [Ni(C_63_H_31_F_10_N_5_S_2_)]·*x*CH_2_Cl_2_ (*x* > 1/2), consists of Ni–porphyrin complexes that are located in general positions and di­chloro­methane solvent mol­ecules that are disordered around centers of inversion. The Ni^II^ ions are in a square-pyramidal (CN5) coordination, with four porphyrin N atoms in the equatorial and a pyridine N atom in the apical position and are shifted out of the porphyrine N_4_ plane towards the coordinating pyridine N atom. The pyridine substituent is not exactly perpendicular to the N_4_ plane with an angle of inter­section between the planes planes of 80.48 (6)°. The di­chloro­methane solvent mol­ecules are hydrogen bonded to one of the four porphyrine N atoms. Two complexes are linked into dimers by two symmetry-equivalent C—H⋯S hydrogen bonds. These dimers are closely packed, leading to cavities in which additional di­chloro­methane solvent mol­ecules are embedded. These solvent mol­ecules are disordered and because no reasonable split model was found, the data were corrected for disordered solvent using the *PLATON* SQUEEZE routine [Spek (2015 ▸). *Acta Cryst.* C**71**, 9–18].

## Abstract 2

The crystal structure of the title compound consists of discrete complexes with a five-coordinate Ni cation and intra­molecular hydrogen-bonded di­chloro­methane solvent mol­ecules that are linked into dimers *via* pairs of inter­molecular C—H⋯S hydrogen bonds.

## Chemical context   

**Figure 1:**
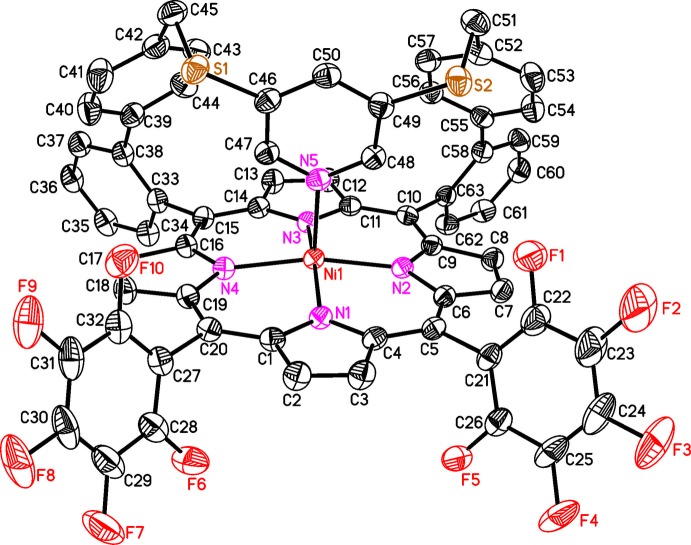
Mol­ecular structure of the title compound with the atom labelling and displacement ellipsoids drawn at the 50% probability level. The H atoms and the solvent mol­ecules are omitted for clarity.

Nickelporphyrins and their axial coordination have been studied from a number of different viewpoints over the last six decades. Their rich coordination behaviour (Caughey *et al.*, 1962 ▸; McLees & Caughey, 1968 ▸; Walker *et al.* 1975 ▸), conformations (Jia *et al.*, 1998 ▸) and photophysics (Kim *et al.*, 1983 ▸; Kim & Holten, 1983 ▸) has attracted inter­est in different fields, including as model compounds for the F430 cofactor (Renner *et al.*, 1991 ▸) or heme (Jentzen *et al.*, 1995 ▸), for applications in solar energy conversion (Shelby *et al.*, 2014 ▸), in hydrogen-evolution (Han *et al.*, 2016 ▸) or redox catalysis (Eom *et al.*, 1997 ▸) and as responsive MRI contrast agents (Venkataramani *et al.*, 2011 ▸; Dommaschk *et al.*, 2014*a* ▸,*b* ▸, 2015*a* ▸,*b* ▸). Square-planar [coordination number (CN) 4] nickelporphyrins are diamagnetic, (*S* = 0), low-spin (LS) complexes. Upon coordination of one (CN5) or two (CN6) axial ligands such as pyridine or piperidine, the nickel cation undergoes spin transition to the high-spin (HS) state. This coordination-induced spin-state switch (CISSS) leads to a drastic change in the spectra and properties of the HS complexes. The coordination and decoordination of the axial ligands in solution is a fast dynamic equilibrium (Kadish *et al.*, 2000 ▸). Thus, the observed properties are dependent on the speciation in the equilibrium defined by the association constants (*K*_1S_, *K*_2_; Thies *et al.*, 2010 ▸). In these equilibria, the dominating species are the CN4 and CN6 complexes, with the CN5 species only formed by up to 10% of porphyrins in solution (Kruglik *et al.*, 2003 ▸). Thus, the characterization of CN5 nickelporphyrins was restricted to transient UV–vis (Kim *et al.*, 1983 ▸) and resonance Raman measurements (Findsen *et al.*, 1986 ▸; Kim *et al.*, 1986 ▸) so far. Recently, the first exclusively five-coordinate (CN5) nickel porphyrin in solution, including its structure in the crystal phase, were presented (Gutzeit *et al.*, 2019 ▸), offering a new approach towards afore-mentioned applications. The axial ligand of the CN5 porphyrin is held in the coordination position by a rigid strap, inducing conformation-dependent spin-state switching. Similar strapped nickelporphyrins showed incomplete axial coordination in solution (Köbke *et al.*, 2019 ▸). The title compound (Fig. 1 ▸) was obtained as a byproduct in the synthesis of a CN5 porphyrin with a similar structure (Gutzeit *et al.*, 2019 ▸) and was metallated under standard conditions. Preorientation of the ligand by the ligand-holding strap should favour Ni coordination. However, ^1^H NMR spectropscopy (500 MHz, CDCl_3_, 298 K) indicates incomplete intra­molecular coordination (82% CN5 HS, 18% CN4 LS) of the title compound. One application is pH measurements in non-aqueous solutions because coordination and NMR signals are dependent on the protonation state of the pyridine moiety. The NMR spectra revealed an unexpected behaviour of the title compound, because the geminal coupling of the CH_2_-protons indicates confined movement of the pyridine moiety and hindered ring inversion of the strap (see Figure S1 in the supporting information).
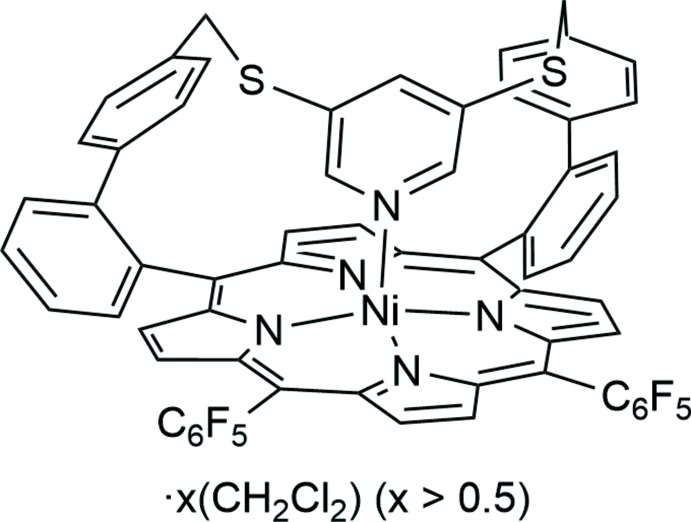


## Structural commentary   

**Figure 4:**
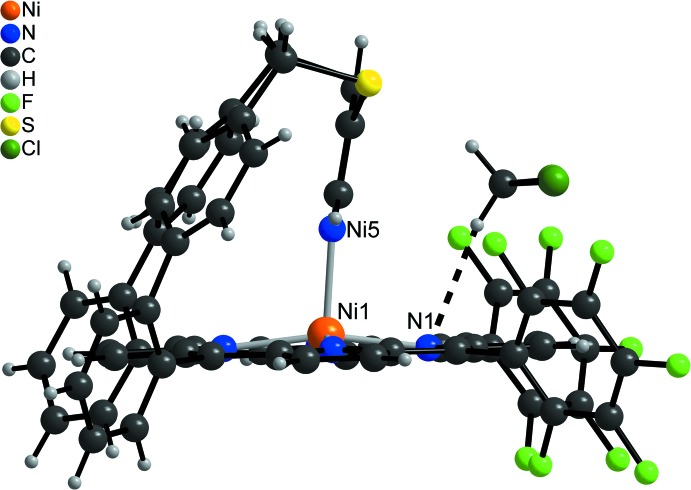
Side view of the complex showing the orientation of the pyridine ring relative to the N_4_ plane. The inter­molecular hydrogen bond is shown as dashed line and the disorder of the di­chloro­methane mol­ecule is omitted for clarity.

In the crystal structure of the title compound, [Ni(C_63_H_31_F_10_N_5_S_2_)]·*x*CH_2_Cl_2_ (*x* > 1/2), the Ni^II^ ions are coordinated by the four N atoms of the porphyrine moiety within a square-planar ligand field and the Ni coordination is completed by a pyridine N atom in the apical position, leading to a square-pyramidal coordination environment (CN5) (Figs. 1 ▸–3 ▸ ▸). The porphyrine ring plane is not fully planar with maximum deviations of the C atoms from the mean plane of 0.137 (3) Å. The Ni cation is shifted by 0.250 (3) Å out of the N_4_ plane towards the coordinating pyridine N atom (Fig. 4 ▸). The Ni—N bond lengths (Table 1 ▸) to the porphyrine N atoms ranges from 2.0350 (17) to 2.0434 (17) Å and are in agreement with values retrieved from literature, indicating that the Ni^II^ ion is in the high-spin state (Thies *et al.*, 2010 ▸). The Ni—N bond length to the pyridine N atom of 2.1122 (17) Å is significantly longer and agrees well with the 2.11 Å that are observed in the CN5 porphyrin (Gutzeit *et al.*, 2019 ▸). Compared to octa­hedral (CN6) nickelporphyrins with two axial pyridine ligands, the Ni—N distance is shortened by ∼0.10 Å (Thies *et al.*, 2010 ▸). The pyridine ring is not exactly perpendicular to the N4 plane (Fig. 4 ▸), the angle of inter­section between them amounting to 80.48 (6)°, in good agreement with similar complexes (Thies *et al.*, 2010 ▸). The tetra­fluoro­phenyl rings are rotated out of the N_4_ plane by 67.43 (5) and 68.74 (6)°, and the phenyl rings (C39–C44 and C58–C63) by 58.82 (6) and 72.59 (5)°, respectively. The dihedral angles between the biphenyl units amount to 63.02 (9) and 53.45 (8)°.

## Supra­molecular features   

**Figure 5:**
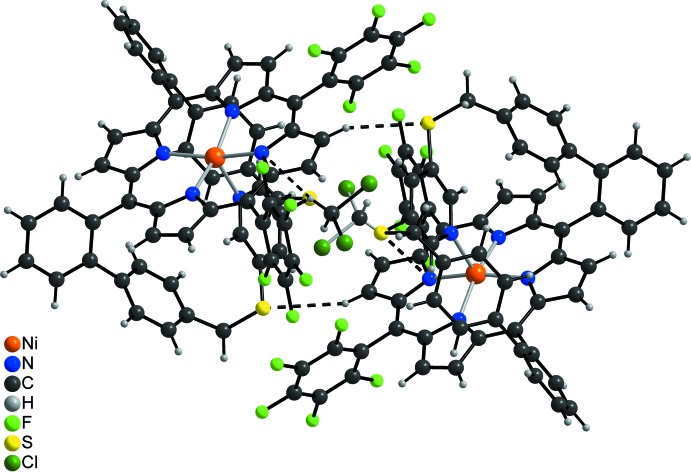
Crystal packing of the title compound with a view of a centrosymmetric dimer with inter­molecular hydrogen bonding shown as dashed lines. The two orientations of the disordered di­chloro­methane mol­ecule are shown with black and grey bonds.

In the crystal structure of the title compound, the discrete Ni porphyrine complexes are linked into dimers *via* centrosymmetric pairs of inter­molecular C—H⋯S hydrogen bonds between the porphyrine H atoms and the sulfur atoms (Fig. 5 ▸ and Table 2 ▸). Between the dimers, cavities are formed that are occupied by the di­chloro­methane solvent mol­ecules, which are disordered about centers of inversion. These solvent mol­ecules are linked by inter­molecular C—H⋯Cl hydrogen bonding to the nitro­gen atom N1 of the porphyrine unit that is not shielded by the strap (Fig. 5 ▸). The C—H⋯S angle is close to linearity, indicating that this is a relatively strong inter­action (Table 2 ▸). The dimeric units are packed in such a way that cavities are formed in which additional, completely disordered dichlormethane solvent mol­ecules are embedded, for which no reasonable structure model was found.

## Database survey   

According to a search of the Cambridge Structural Database (CSD, Version 5.40, update of February 2019; Groom *et al.*, 2016 ▸), axial coordination of metal porphyrins is highly metal dependent. Two examples of CN5 nickelporphyrins are known that have been characterized by single-crystal structure analysis (Kumar & Sankar, 2014 ▸; Gutzeit *et al.*, 2019 ▸), while zinc porphyrins almost exclusively form CN5 complexes (Paul *et al.*, 2003 ▸; Deutman *et al.*, 2014 ▸). The application of strapped porphyrins for controlling axial coordination is an established approach (Richard *et al.*, 1998 ▸) for mimicking heme complexes (Hijazi *et al.*, 2010 ▸; Melin *et al.*, 2012 ▸; Zhou *et al.*, 2012 ▸). With nickel(II) porphyrins with nitro­gen-containing ligands almost exclusively form CN4 (Nurco *et al.*, 2002 ▸; Halime *et al.*, 2007 ▸; Bediako *et al.*, 2014 ▸) or CN6 (Thies *et al.*, 2010 ▸; Dommaschk *et al.*, 2014*b* ▸) complexes, in rare cases a CN6 complex is formed with oxygen-containing ligands (Ozette *et al.*, 1997 ▸).

## Synthesis and crystallization   

The freebase porphyrin of the title compound was obtained as a byproduct of a variant of the published procedure (Gutzeit *et al.*, 2019 ▸). The compound is synthesized from a linked di­aldehyde under acidic conditions through macrocycle condensation with penta­fluoro­phenyl­dipyrro­methane. The reaction was performed under reflux for 17 h before the addition of 2,3-dichloro-5,6-dicyano-1,4-benzoquinone (DDQ). At elevated temperatures, the scrambling mechanism, acidic cleavage and rearrangement of oligopyrrols dominates the product formation, leading to the 5,10-bridged scrambling porphyrin of the title compound. The freebase porphyrins were separated by column chromatography (silica gel, di­chloro­methane and silica gel, toluene) and precipitated from di­chloro­methane by diffusion of methanol (89 mg, 4.3%).

^1^H NMR (600 MHz, CDCl_3_, 298 K, TMS): δ = 8.95 (*s*, 2 H, *H*_β_), 8.65 (*d*, ^3^ *J* = 4.5 Hz, 2 H, *H*_β_), 8.63 (*s*, 2 H, *H*_β_), 8.54 (*d*, ^3^ *J* = 4.5 Hz, 2 H, *H*_β_), 8.28 (*dd*, ^3^ *J* = 7.4 Hz, ^4^ *J* = 1.0 Hz, 2 H, *H*-6′), 7.90 (*td*, ^3^ *J* = 7.8 Hz, ^4^ *J* = 1.3 Hz, 2 H, *H*-4′), 7.80 (*dd*, ^3^ *J* = 7.9 Hz, ^4^ *J* = 1.0 Hz, 2 H, *H*-3′), 7.75 (*td*, ^3^ *J* = 7.5 Hz, ^4^ *J* = 1.3 Hz, 2 H, *H*-5′), 7.29 (*s*, 1 H, *H*-4′′′), 6.68 (*d*, ^3^ *J* = 8.2 Hz, 4 H, *H*-2′′), 5.81 (*d*, ^3^ *J* = 8.2 Hz, 4 H, *H*-3′′), 3.18 (*d*, ^2^ *J* = 14.6 Hz, 2 H, C*H*_2,a_), 3.05 (*d*, ^2^ *J* = 14.6 Hz, 2 H, C*H*_2,b_), −2.80 (*s*, 2 H, N*H*) ppm. Unobserved signals: *H*-2′′′. ^13^C NMR (151 MHz, CDCl_3_, 298 K, TMS): δ = 151.8 (*C*3′′′), 145.1 (*C*4′′′), 144.7 (*C*2′), 140.4 (*C*1′′), 139.9 (*C*1′), 135.7 (*C*4′′), 134.7 (*C*6′), 129.4 (*C*2′′), 129.3 (*C*3′), 129.2 (*C*4′), 127.3 (*C*3′′), 125.9 (*C*5′), 121.4 (*C*5, *C*10), 101.1 (*C*15, *C*20), 40.5 (*C*H_2_) ppm. Unobserved signals: *C*2′′′, *C*_α_, *C*_β_, *C*_6_F_5_. ^19^F NMR (471 MHz, CDCl_3_, 298 K): δ = −136.96 (*dd*, ^3^ *J* = 24.4 Hz, ^4^ *J* = 7.8 Hz, *F*-*ortho*), −137.27 (*dd*, ^3^ *J* = 24.0 Hz, ^4^ *J* = 7.8 Hz, *F*-*ortho*), −153.03 (*t*, ^3^ *J* = 21.0 Hz, *F*-*para*), −(162.35–162.62) (*m*, *F*-*meta*) ppm. FT–IR (ATR): ν = 3310.3 (*w*), 3026.7 (*w*), 1650.5 (*w*), 1519.6 (*s*), 1496.0 (*vs*), 1474.9 (*s*), 1440.4 (*m*), 1393.7 (*m*), 1349.2 (*m*), 1266.0 (*w*), 1126.8 (*w*), 1042.1 (*m*), 975.3 (*vs*), 971.2 (*s*), 917.3 (*vs*), 882.7 (*m*), 837.3 (*m*), 800.1 (*vs*), 762.9 (*vs*), 746.9 (*vs*), 713.0 (*s*), 701.2 (*vs*), 664.7 (*s*), 650.2 (*s*), 638.3 (*m*), 598.1 (*m*), 553.9 (*m*), 529.6 (*m*), 505.2 (*m*), 460.0 (*w*), 430.2 (*w*), 407.2 (*m*) cm^−1^. MS (EI): *m*/*z* (%) = 1113.20 (100) [*M*]^+^, 556.59 (13) [*M*]^2+^ u. HRMS (EI) calculated for C_63_H_33_F_10_N_5_S_2_: 1113.2018 u, found: 1113.2023 u, dif.: 0.5 ppm.

The nickel cation was introduced under standard conditions (20 mg porphyrin, 80 mg Ni(acac)_2_, 15 mL toluene, reflux, 23 h) followed by filtration through a silica plug (di­chloro­methane) (21 mg, 99%). Single crystals were obtained by dissolving the compound in di­chloro­methane and gas phase diffusion of methanol.

M.p. > 673 K. Decomposition starting from 600 K. ^1^H NMR (600 MHz, CDCl_3_, 300 K, TFA): δ = 8.68 (*s*, 6 H, *H*_β_), 8.54 (*s*, 2 H, *H*_β_), 8.05 (*d*, ^3^ *J* = 7.4 Hz, 2 H, *H*-6′), 7.90–7.84 (*m*, 3 H, *H*-4′, *H*-4′′′), 7.79 (*d*, ^3^ *J* = 7.9 Hz, 2 H, *H*-3′), 7.72 (*t*, ^3^ *J* = 7.5 Hz, 2 H, *H*-5′), 7.52 (*d*, ^4^ *J* = 1.3 Hz, 2 H, *H*-2′′′), 6.73 (*d*, 3*J* = 8.3 Hz, 4 H, *H*-2′′), 6.39 (*d*, ^3^ *J* = 8.3 Hz, 4 H, *H*-3′′), 3.61 (*d*, ^2^ *J* = 14.9 Hz, 2 H, C*H*_2,a_), 3.56 (*d*, ^2^ *J* = 14.9 Hz, 2 H, C*H*_2,b_) ppm. ^13^C NMR (151 MHz, CDCl_3_, 300 K, TFA): δ = 145.8 (*C*4′′′), 143.1 (*C*2′), 141.6 (*C*1′′), 140.7 (*C*3′′′), 138.5 (*C*1′), 137.5 (*C*2′′′), 135.0 (*C*6′), 134.2 (*C*_β_), 133.3 (*C*_β_), 132.9 (*C*4′′), 131.6 (*C*_β_), 130.6 (*C*_β_), 129.8 (*C*2′′), 129.6 (*C*3′), 129.6 (*C*4′), 128.0 (*C*3′′), 126.6 (*C*5′), 38.4 (*C*H_2_) ppm. Unobserved signals: C_*meso*_, *C*_α_, *C*_6_F_5_. ^19^F NMR (471 MHz, CDCl_3_, 300 K, TFA): δ = −137.04 (*dd*, ^3^ *J* = 23.6 Hz, ^4^ *J* = 7.4 Hz, *F*-*ortho*), −138.11 (*dd*, ^3^ *J* = 23.6 Hz, ^4^ *J* = 6.3 Hz, *F*-*ortho*), −152.14 *(t*, ^3^ *J* = 20.6 Hz, *F*-*para*), −161.67 (*td*, ^3^ *J* = 22.0 Hz, ^4^ *J* = 8.3 Hz, *F*-*meta*), −162.01 (*td*, ^3^ *J* = 22.2 Hz, ^4^ *J* = 8.3 Hz, *F*-*meta*) ppm. FT–IR (ATR): ν = 3023.7 (*w*), 2920.4 (*w*), 2843.3 (*w*), 2748.1 (*w*), 1685.6 (*s*), 1595.8 (*m*), 1517.3 (*m*), 1477.2 (*s*), 1440.8 (*m*), 1390.9 (*m*), 1339.2 (*m*), 1297.2 (*m*), 1254.9 (*m*), 1177.5 (*m*), 1072.9 (*m*), 984.0 (*vs*), 949.2 (*s*), 930.4 (*m*), 832.6 (*s*), 815.7 (*m*), 798.9 (*m*), 752.5 (*vs*), 729.9 (*s*), 700.8 (*vs*), 655.2 (*m*), 535.4 (*m*), 464.7 (*m*), 441.2 (*m*), 416.9 (*m*) cm^−1^. MS (EI): *m*/*z* (%) = 1169.16 (100) [*M*]^+^, 1027.11 (5) [*M* - C_5_H_4_NS_2_]^+^, 584.54 (12) [*M*]^2+^ u. HRMS (EI) calculated for C_63_H_31_F_10_N_5_NiS_2_: 1169.1215 u, found: 1169.1159 u, dif.: 4.7 ppm.

## Refinement   

Crystal data, data collection and structure refinement details are summarized in Table 3 ▸. The C—H hydrogen atoms were located in difference-Fourier maps but were positioned with idealized geometry and refined with isotropic with *U*_iso_(H) = 1.2*U*_eq_(C) using a riding model. After structure refinement using a model with one Ni porphyrine complex and a half di­chloro­methane solvent mol­ecule disordered about a center of inversion, there was significant residual electron density that definitely corresponded to an additional di­chloro­methane mol­ecule that was disordered over several orientations. A number of different split models were tried, using restraints for the geometry and for the components of the anisotropic displacement parameters, but no reasonable structure model was found and very large anisotropic displacement parameters were obtained. Therefore, the contribution of this solvent to the electron density was removed with the SQUEEZE (Spek, 2015 ▸) routine in *PLATON*, which leads to a reasonable structure model and very good reliability factors. Their formula mass and unit-cell characteristics were not taken into account during refinement. By this procedure, the amount of di­chloro­methane cannot be determined accurately and there is indication that this position is not fully occupied, which is highly likely because this solvent is very unstable and starts to decompose during the sample preparation.

## Supplementary Material

Crystal structure: contains datablock(s) I. DOI: 10.1107/S2056989019009836/lh5902sup1.cif

Structure factors: contains datablock(s) I. DOI: 10.1107/S2056989019009836/lh5902Isup2.hkl

Fig. S1. 1H NMR spectrum of the title compound and its 5,15-strapped isomer showing the splitting of the methylene protons due to geminal coupling. DOI: 10.1107/S2056989019009836/lh5902sup3.tif

CCDC reference: 1942625

Additional supporting information:  crystallographic information; 3D view; checkCIF report

## Figures and Tables

**Figure 2:**
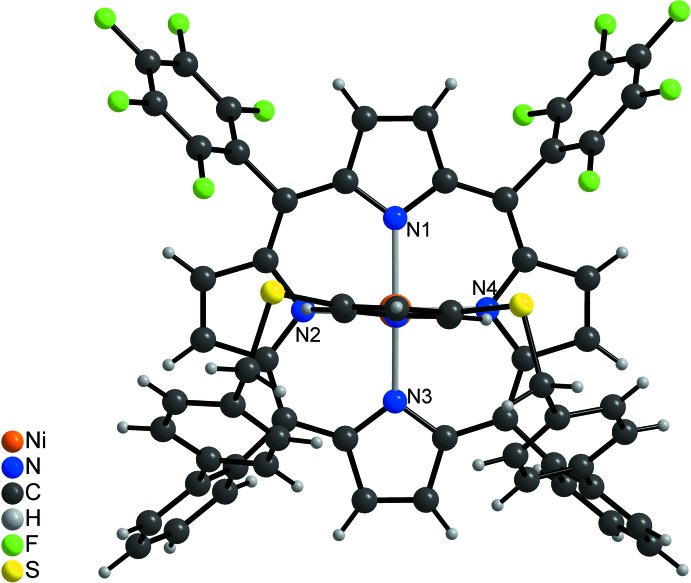
Mol­ecular structure of the title compound in a view onto the porphyrin plane.

**Figure 3:**
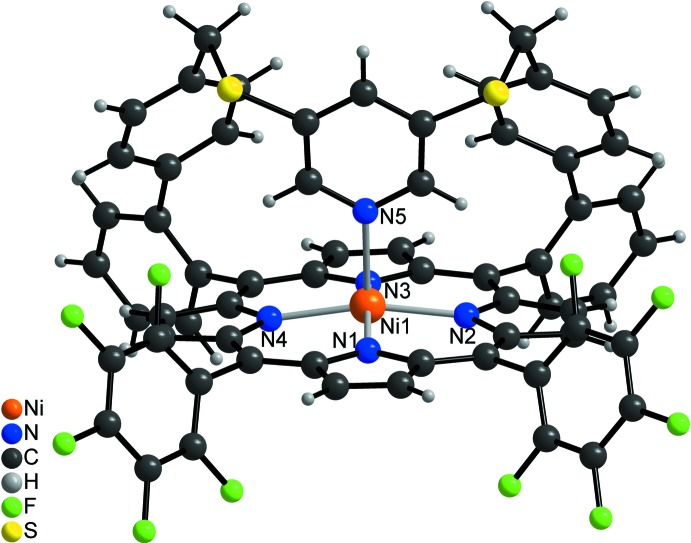
Mol­ecular structure of the title compound with view of the Ni coordination.

**Table 1:** Selected geometric parameters (Å, °)

Ni1—N4	2.0350 (17)	Ni1—N2	2.0434 (17)
Ni1—N3	2.0402 (17)	Ni1—N5	2.1122 (17)
Ni1—N1	2.0407 (17)		
			
N4—Ni1—N3	89.66 (7)	N1—Ni1—N2	89.29 (7)
N4—Ni1—N1	89.03 (7)	N4—Ni1—N5	96.84 (7)
N3—Ni1—N1	166.05 (7)	N3—Ni1—N5	100.53 (7)
N4—Ni1—N2	165.76 (7)	N1—Ni1—N5	93.42 (7)
N3—Ni1—N2	88.58 (7)	N2—Ni1—N5	97.37 (7)

**Table 2:** Hydrogen-bond geometry (Å, °)

*D*—H⋯*A*	*D*—H	H⋯*A*	*D*⋯*A*	*D*—H⋯*A*
C2—H2⋯S2^i^	0.95	3.02	3.886 (2)	153
C71—H71*B*⋯N1^i^	0.96	2.61	3.555 (8)	169

Symmetry code: (i) 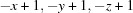.

**Table 3:** Experimental details

Crystal data	
Chemical formula	[Ni(C_63_H_31_F_10_N_5_S_2_)]·0.5CH_2_Cl_2_
*M*_r_	1213.22
Crystal system, space group	Monoclinic, *P*2_1_/*c*
Temperature (K)	170
*a*, *b*, *c* (Å)	14.0919 (3), 22.0127 (4), 17.9648 (3)
β (°)	93.950 (1)
*V* (Å^3^)	5559.46 (18)
*Z*	4
Radiation type	Mo *K*α
μ (mm^−1^)	0.55
Crystal size (mm)	0.12 × 0.10 × 0.07

Data collection	
Diffractometer	Stoe *IPDS2*
Absorption correction	Numerical (*X-RED* and *X-SHAPE*; Stoe & Cie, 2008 ▸)
*T*_min_, *T*_max_	0.855, 0.932
No. of measured, independent and observed [*I* > 2σ(*I*)] reflections	55497, 12087, 10733
*R*_int_	0.041
(sin θ/λ)_max_ (Å^−1^)	0.639

Refinement	
*R*[*F* ^2^ > 2σ(*F* ^2^)], *wR*(*F* ^2^), *S*	0.044, 0.129, 1.05
No. of reflections	12087
No. of parameters	758
H-atom treatment	H-atom parameters constrained
Δρ_max_, Δρ_min_ (e Å^−3^)	0.66, −1.08

Computer programs: *X-AREA* (Stoe & Cie, 2008 ▸), *SHELXT* (Sheldrick, 2015*a* ▸), *SHELXL2014* (Sheldrick, 2015*b* ▸), *XP* (Sheldrick, 2008 ▸), *DIAMOND* (Brandenburg, 2014 ▸) and *publCIF* (Westrip, 2010 ▸).

## supplementary crystallographic information

### Crystal data

**Table:**

[Ni(C_63_H_31_F_10_N_5_S_2_)]·0.5CH_2_Cl_2_	*F*(000) = 2460
*M**_r_* = 1213.22	*D*_x_ = 1.449 Mg m^−^^3^
Monoclinic, *P*2_1_/*c*	Mo *K*α radiation, λ = 0.71073 Å
*a* = 14.0919 (3) Å	Cell parameters from 55528 reflections
*b* = 22.0127 (4) Å	θ = 1.5–27.0°
*c* = 17.9648 (3) Å	µ = 0.55 mm^−^^1^
β = 93.950 (1)°	*T* = 170 K
*V* = 5559.46 (18) Å^3^	Block, red
*Z* = 4	0.12 × 0.10 × 0.07 mm

### Data collection

**Table:**

Stoe IPDS-2 diffractometer	10733 reflections with *I* > 2σ(*I*)
ω scans	*R*_int_ = 0.041
Absorption correction: numerical (X-RED and X-SHAPE; Stoe & Cie, 2008)	θ_max_ = 27.0°, θ_min_ = 1.5°
*T*_min_ = 0.855, *T*_max_ = 0.932	*h* = −18→18
55497 measured reflections	*k* = −25→28
12087 independent reflections	*l* = −22→22

### Refinement

**Table:**

Refinement on *F*^2^	0 restraints
Least-squares matrix: full	Hydrogen site location: mixed
*R*[*F*^2^ > 2σ(*F*^2^)] = 0.044	H-atom parameters constrained
*wR*(*F*^2^) = 0.129	*w* = 1/[σ^2^(*F*_o_^2^) + (0.0758*P*)^2^ + 3.7723*P*] where *P* = (*F*_o_^2^ + 2*F*_c_^2^)/3
*S* = 1.05	(Δ/σ)_max_ = 0.002
12087 reflections	Δρ_max_ = 0.66 e Å^−^^3^
758 parameters	Δρ_min_ = −1.07 e Å^−^^3^

### Special details

**Table:**

Geometry. All esds (except the esd in the dihedral angle between two l.s. planes) are estimated using the full covariance matrix. The cell esds are taken into account individually in the estimation of esds in distances, angles and torsion angles; correlations between esds in cell parameters are only used when they are defined by crystal symmetry. An approximate (isotropic) treatment of cell esds is used for estimating esds involving l.s. planes.

### Fractional atomic coordinates and isotropic or equivalent isotropic displacement parameters (Å^2^)

**Table:**

	*x*	*y*	*z*	*U*_iso_*/*U*_eq_	Occ. (<1)
Ni1	0.58798 (2)	0.71647 (2)	0.57642 (2)	0.02141 (8)	
N1	0.59348 (12)	0.63329 (8)	0.62668 (9)	0.0245 (3)	
N2	0.46308 (12)	0.73353 (8)	0.62395 (9)	0.0230 (3)	
N3	0.59565 (12)	0.80659 (8)	0.55101 (9)	0.0233 (3)	
N4	0.72676 (12)	0.70653 (8)	0.55409 (10)	0.0239 (3)	
C1	0.66429 (15)	0.59083 (9)	0.62313 (12)	0.0269 (4)	
C2	0.63488 (17)	0.53424 (10)	0.65507 (13)	0.0334 (5)	
H2	0.6708	0.4977	0.6592	0.040*	
C3	0.54726 (17)	0.54296 (10)	0.67780 (13)	0.0337 (5)	
H3	0.5092	0.5137	0.7008	0.040*	
C4	0.52167 (15)	0.60528 (9)	0.66069 (11)	0.0266 (4)	
C5	0.43626 (15)	0.63265 (9)	0.67759 (11)	0.0267 (4)	
C6	0.41063 (14)	0.69294 (9)	0.66208 (11)	0.0249 (4)	
C7	0.32499 (16)	0.72149 (10)	0.68415 (12)	0.0306 (4)	
H7	0.2770	0.7031	0.7112	0.037*	
C8	0.32589 (15)	0.77925 (10)	0.65907 (12)	0.0301 (4)	
H8	0.2786	0.8093	0.6651	0.036*	
C9	0.41223 (14)	0.78671 (9)	0.62136 (11)	0.0244 (4)	
C10	0.43930 (14)	0.84098 (9)	0.58821 (11)	0.0244 (4)	
C11	0.52614 (14)	0.84960 (9)	0.55633 (11)	0.0246 (4)	
C12	0.55625 (16)	0.90652 (10)	0.52642 (13)	0.0306 (4)	
H12	0.5203	0.9430	0.5223	0.037*	
C13	0.64549 (16)	0.89808 (10)	0.50525 (13)	0.0311 (4)	
H13	0.6846	0.9277	0.4842	0.037*	
C14	0.66991 (14)	0.83565 (9)	0.52072 (11)	0.0255 (4)	
C15	0.75732 (14)	0.80948 (9)	0.50740 (12)	0.0264 (4)	
C16	0.78312 (14)	0.74944 (9)	0.52339 (12)	0.0257 (4)	
C17	0.87512 (16)	0.72371 (10)	0.51174 (14)	0.0327 (5)	
H17	0.9270	0.7440	0.4914	0.039*	
C18	0.87370 (16)	0.66555 (10)	0.53515 (14)	0.0322 (5)	
H18	0.9241	0.6370	0.5341	0.039*	
C19	0.78106 (14)	0.65502 (9)	0.56210 (12)	0.0265 (4)	
C20	0.75174 (15)	0.60063 (9)	0.59340 (12)	0.0266 (4)	
C21	0.36750 (16)	0.59433 (10)	0.71663 (12)	0.0303 (4)	
C22	0.2819 (2)	0.57573 (13)	0.68182 (15)	0.0436 (6)	
C23	0.2180 (2)	0.53996 (16)	0.71763 (19)	0.0602 (8)	
C24	0.2399 (3)	0.52226 (14)	0.79003 (19)	0.0587 (8)	
C25	0.3250 (2)	0.53948 (12)	0.82664 (15)	0.0459 (6)	
C26	0.38710 (17)	0.57540 (10)	0.78984 (13)	0.0341 (5)	
F1	0.25770 (12)	0.59288 (9)	0.61162 (9)	0.0581 (5)	
F2	0.13568 (17)	0.52346 (14)	0.68200 (14)	0.0979 (9)	
F3	0.17864 (18)	0.48761 (11)	0.82540 (15)	0.0909 (8)	
F4	0.34636 (15)	0.52264 (8)	0.89743 (10)	0.0620 (5)	
F5	0.46837 (11)	0.59194 (7)	0.82692 (8)	0.0439 (3)	
C27	0.82125 (15)	0.54910 (10)	0.59874 (13)	0.0305 (4)	
C28	0.86257 (17)	0.53054 (11)	0.66689 (15)	0.0385 (5)	
C29	0.92457 (19)	0.48197 (13)	0.67457 (18)	0.0495 (7)	
C30	0.94680 (18)	0.45035 (12)	0.6117 (2)	0.0512 (7)	
C31	0.90800 (18)	0.46779 (11)	0.54311 (18)	0.0445 (6)	
C32	0.84641 (17)	0.51655 (10)	0.53690 (15)	0.0358 (5)	
F6	0.84195 (12)	0.56018 (8)	0.72897 (9)	0.0525 (4)	
F7	0.96200 (14)	0.46548 (11)	0.74156 (13)	0.0752 (6)	
F8	1.00598 (13)	0.40282 (8)	0.61798 (15)	0.0765 (7)	
F9	0.92962 (13)	0.43661 (8)	0.48228 (12)	0.0625 (5)	
F10	0.81040 (12)	0.53211 (7)	0.46861 (9)	0.0469 (4)	
C33	0.83157 (14)	0.84931 (9)	0.47646 (13)	0.0281 (4)	
C34	0.88260 (16)	0.88914 (10)	0.52414 (14)	0.0329 (5)	
H34	0.8662	0.8931	0.5743	0.040*	
C35	0.95687 (16)	0.92312 (10)	0.49967 (15)	0.0362 (5)	
H35	0.9911	0.9502	0.5328	0.043*	
C36	0.98110 (16)	0.91751 (11)	0.42641 (15)	0.0375 (5)	
H36	1.0326	0.9403	0.4094	0.045*	
C37	0.93014 (16)	0.87869 (11)	0.37827 (14)	0.0358 (5)	
H37	0.9468	0.8753	0.3281	0.043*	
C38	0.85461 (15)	0.84444 (10)	0.40201 (13)	0.0309 (4)	
C39	0.80068 (16)	0.80206 (11)	0.35035 (13)	0.0326 (5)	
C40	0.84598 (18)	0.75224 (13)	0.32051 (16)	0.0447 (6)	
H40	0.9131	0.7483	0.3283	0.054*	
C41	0.79485 (19)	0.70860 (14)	0.27980 (17)	0.0473 (6)	
H41	0.8268	0.6744	0.2611	0.057*	
C42	0.69674 (18)	0.71422 (12)	0.26586 (13)	0.0360 (5)	
C43	0.65239 (18)	0.76588 (12)	0.29106 (14)	0.0384 (5)	
H43	0.5862	0.7717	0.2794	0.046*	
C44	0.70348 (18)	0.80909 (11)	0.33306 (14)	0.0381 (5)	
H44	0.6718	0.8440	0.3503	0.046*	
C45	0.64009 (18)	0.66380 (12)	0.22885 (13)	0.0389 (5)	
H45A	0.6730	0.6490	0.1854	0.047*	
H45B	0.5771	0.6796	0.2103	0.047*	
S1	0.62357 (4)	0.60005 (3)	0.29283 (3)	0.03453 (13)	
C46	0.54694 (16)	0.63273 (10)	0.35631 (12)	0.0292 (4)	
C47	0.58379 (15)	0.66201 (9)	0.42082 (11)	0.0273 (4)	
H47	0.6505	0.6684	0.4274	0.033*	
N5	0.52841 (12)	0.68159 (8)	0.47408 (9)	0.0257 (3)	
C48	0.43431 (15)	0.67159 (10)	0.46450 (12)	0.0278 (4)	
H48	0.3950	0.6848	0.5022	0.033*	
C49	0.39175 (15)	0.64277 (10)	0.40170 (13)	0.0302 (4)	
C50	0.44935 (16)	0.62400 (10)	0.34651 (12)	0.0307 (4)	
H50	0.4222	0.6053	0.3024	0.037*	
S2	0.26911 (4)	0.62465 (3)	0.39663 (3)	0.03449 (13)	
C51	0.21774 (18)	0.69105 (12)	0.34671 (14)	0.0378 (5)	
H51A	0.2489	0.6964	0.2994	0.045*	
H51B	0.1491	0.6840	0.3342	0.045*	
C52	0.23014 (16)	0.74738 (11)	0.39249 (13)	0.0338 (5)	
C53	0.16432 (17)	0.76351 (12)	0.44322 (15)	0.0396 (5)	
H53	0.1073	0.7407	0.4451	0.047*	
C54	0.18041 (17)	0.81234 (12)	0.49108 (14)	0.0376 (5)	
H54	0.1339	0.8231	0.5246	0.045*	
C55	0.26431 (15)	0.84586 (10)	0.49051 (12)	0.0293 (4)	
C56	0.32876 (16)	0.83131 (10)	0.43773 (12)	0.0309 (4)	
H56	0.3851	0.8547	0.4352	0.037*	
C57	0.31159 (17)	0.78307 (11)	0.38893 (13)	0.0328 (5)	
H57	0.3557	0.7742	0.3527	0.039*	
C58	0.28648 (15)	0.89443 (10)	0.54606 (12)	0.0281 (4)	
C59	0.22139 (16)	0.94130 (11)	0.55572 (13)	0.0335 (5)	
H59	0.1627	0.9417	0.5264	0.040*	
C60	0.24100 (17)	0.98704 (11)	0.60726 (14)	0.0355 (5)	
H60	0.1957	1.0182	0.6135	0.043*	
C61	0.32663 (18)	0.98732 (10)	0.64973 (13)	0.0343 (5)	
H61	0.3409	1.0192	0.6844	0.041*	
C62	0.39173 (16)	0.94075 (10)	0.64144 (12)	0.0307 (4)	
H62	0.4505	0.9410	0.6707	0.037*	
C63	0.37196 (14)	0.89351 (9)	0.59075 (11)	0.0253 (4)	
C71	0.4950 (5)	0.4754 (3)	0.5071 (4)	0.0610 (16)	0.5
H71A	0.5180	0.4593	0.5547	0.073*	0.5
H71B	0.4734	0.4420	0.4760	0.073*	0.5
Cl1	0.58828 (19)	0.51258 (13)	0.46611 (14)	0.0832 (6)	0.5
Cl2	0.39844 (19)	0.52500 (13)	0.51963 (14)	0.0821 (6)	0.5

### Atomic displacement parameters (Å^2^)

**Table:**

	*U*^11^	*U*^22^	*U*^33^	*U*^12^	*U*^13^	*U*^23^
Ni1	0.02354 (13)	0.01875 (13)	0.02204 (13)	−0.00082 (9)	0.00226 (9)	0.00049 (9)
N1	0.0272 (8)	0.0212 (8)	0.0254 (8)	0.0005 (6)	0.0035 (6)	0.0012 (6)
N2	0.0256 (8)	0.0204 (8)	0.0232 (8)	−0.0005 (6)	0.0024 (6)	0.0016 (6)
N3	0.0242 (8)	0.0214 (8)	0.0244 (8)	−0.0011 (6)	0.0026 (6)	0.0001 (6)
N4	0.0253 (8)	0.0198 (8)	0.0267 (8)	−0.0011 (6)	0.0025 (6)	0.0002 (6)
C1	0.0308 (10)	0.0211 (9)	0.0287 (10)	0.0019 (8)	0.0013 (8)	0.0016 (8)
C2	0.0385 (12)	0.0221 (10)	0.0401 (12)	0.0029 (9)	0.0082 (9)	0.0054 (9)
C3	0.0389 (12)	0.0229 (10)	0.0402 (12)	0.0004 (9)	0.0101 (9)	0.0057 (9)
C4	0.0314 (10)	0.0228 (10)	0.0259 (10)	−0.0017 (8)	0.0036 (8)	0.0024 (7)
C5	0.0302 (10)	0.0252 (10)	0.0250 (10)	−0.0024 (8)	0.0042 (8)	0.0029 (8)
C6	0.0258 (9)	0.0255 (10)	0.0237 (9)	−0.0016 (8)	0.0029 (7)	0.0005 (7)
C7	0.0302 (10)	0.0319 (11)	0.0306 (11)	0.0008 (8)	0.0076 (8)	0.0043 (8)
C8	0.0299 (10)	0.0300 (11)	0.0309 (11)	0.0039 (8)	0.0062 (8)	0.0041 (8)
C9	0.0249 (9)	0.0250 (10)	0.0232 (9)	0.0019 (7)	0.0018 (7)	0.0008 (7)
C10	0.0260 (9)	0.0232 (9)	0.0234 (9)	0.0020 (7)	−0.0013 (7)	0.0001 (7)
C11	0.0278 (9)	0.0220 (9)	0.0237 (9)	0.0001 (7)	0.0004 (7)	0.0013 (7)
C12	0.0329 (11)	0.0225 (10)	0.0366 (11)	0.0011 (8)	0.0040 (9)	0.0042 (8)
C13	0.0317 (11)	0.0234 (10)	0.0388 (12)	−0.0007 (8)	0.0067 (9)	0.0049 (8)
C14	0.0282 (10)	0.0219 (9)	0.0266 (9)	−0.0024 (8)	0.0025 (8)	0.0010 (7)
C15	0.0261 (10)	0.0239 (10)	0.0293 (10)	−0.0028 (8)	0.0031 (8)	−0.0004 (8)
C16	0.0246 (9)	0.0229 (10)	0.0297 (10)	−0.0017 (7)	0.0021 (8)	−0.0008 (8)
C17	0.0267 (10)	0.0275 (11)	0.0448 (13)	−0.0012 (8)	0.0090 (9)	0.0010 (9)
C18	0.0274 (10)	0.0262 (10)	0.0436 (12)	0.0014 (8)	0.0059 (9)	0.0008 (9)
C19	0.0263 (9)	0.0238 (10)	0.0294 (10)	0.0010 (8)	0.0020 (8)	−0.0014 (8)
C20	0.0290 (10)	0.0219 (9)	0.0289 (10)	0.0007 (8)	0.0013 (8)	−0.0007 (8)
C21	0.0357 (11)	0.0236 (10)	0.0328 (11)	−0.0006 (8)	0.0111 (9)	0.0038 (8)
C22	0.0456 (14)	0.0458 (14)	0.0400 (13)	−0.0156 (11)	0.0078 (11)	0.0025 (11)
C23	0.0547 (17)	0.063 (2)	0.0640 (19)	−0.0312 (15)	0.0126 (15)	0.0021 (15)
C24	0.070 (2)	0.0452 (16)	0.0652 (19)	−0.0203 (14)	0.0326 (16)	0.0093 (14)
C25	0.0678 (18)	0.0311 (12)	0.0417 (14)	0.0056 (12)	0.0245 (13)	0.0116 (10)
C26	0.0410 (12)	0.0271 (11)	0.0355 (12)	0.0046 (9)	0.0120 (9)	0.0037 (9)
F1	0.0503 (9)	0.0797 (13)	0.0433 (9)	−0.0257 (9)	−0.0047 (7)	0.0082 (8)
F2	0.0737 (14)	0.133 (2)	0.0875 (16)	−0.0721 (15)	0.0073 (12)	0.0028 (15)
F3	0.1011 (17)	0.0806 (15)	0.0967 (17)	−0.0436 (13)	0.0481 (14)	0.0186 (13)
F4	0.0930 (14)	0.0503 (10)	0.0458 (9)	0.0125 (9)	0.0287 (9)	0.0241 (8)
F5	0.0466 (8)	0.0528 (9)	0.0323 (7)	0.0078 (7)	0.0042 (6)	0.0086 (6)
C27	0.0279 (10)	0.0218 (10)	0.0420 (12)	−0.0003 (8)	0.0034 (8)	0.0029 (8)
C28	0.0343 (12)	0.0336 (12)	0.0467 (14)	−0.0003 (9)	−0.0029 (10)	0.0032 (10)
C29	0.0332 (12)	0.0443 (15)	0.0697 (19)	0.0029 (11)	−0.0063 (12)	0.0194 (13)
C30	0.0291 (12)	0.0299 (12)	0.096 (2)	0.0081 (10)	0.0115 (13)	0.0120 (14)
C31	0.0328 (12)	0.0269 (11)	0.0760 (19)	0.0002 (9)	0.0191 (12)	−0.0059 (12)
C32	0.0323 (11)	0.0262 (11)	0.0497 (14)	−0.0013 (9)	0.0091 (10)	0.0000 (9)
F6	0.0595 (10)	0.0560 (10)	0.0401 (8)	0.0052 (8)	−0.0100 (7)	−0.0004 (7)
F7	0.0563 (11)	0.0808 (14)	0.0855 (14)	0.0171 (10)	−0.0172 (10)	0.0336 (12)
F8	0.0470 (10)	0.0427 (10)	0.141 (2)	0.0237 (8)	0.0167 (11)	0.0210 (11)
F9	0.0545 (10)	0.0402 (9)	0.0965 (14)	0.0060 (7)	0.0322 (10)	−0.0182 (9)
F10	0.0563 (9)	0.0444 (8)	0.0410 (8)	0.0055 (7)	0.0109 (7)	−0.0054 (7)
C33	0.0249 (9)	0.0218 (9)	0.0380 (11)	0.0002 (7)	0.0043 (8)	0.0032 (8)
C34	0.0315 (11)	0.0262 (10)	0.0413 (12)	−0.0014 (8)	0.0043 (9)	−0.0009 (9)
C35	0.0298 (11)	0.0248 (10)	0.0539 (14)	−0.0047 (8)	0.0008 (10)	0.0013 (9)
C36	0.0289 (11)	0.0293 (11)	0.0547 (15)	−0.0047 (9)	0.0060 (10)	0.0089 (10)
C37	0.0322 (11)	0.0337 (12)	0.0422 (13)	−0.0013 (9)	0.0075 (9)	0.0097 (10)
C38	0.0274 (10)	0.0260 (10)	0.0395 (12)	−0.0004 (8)	0.0042 (8)	0.0048 (9)
C39	0.0337 (11)	0.0320 (11)	0.0329 (11)	−0.0054 (9)	0.0070 (9)	0.0045 (9)
C40	0.0305 (12)	0.0497 (15)	0.0546 (16)	−0.0042 (11)	0.0073 (11)	−0.0104 (12)
C41	0.0384 (13)	0.0499 (16)	0.0546 (16)	−0.0033 (11)	0.0101 (11)	−0.0168 (13)
C42	0.0382 (12)	0.0426 (13)	0.0275 (11)	−0.0058 (10)	0.0040 (9)	0.0019 (9)
C43	0.0364 (12)	0.0442 (13)	0.0337 (12)	−0.0003 (10)	−0.0032 (9)	0.0056 (10)
C44	0.0394 (12)	0.0348 (12)	0.0395 (13)	0.0014 (10)	−0.0009 (10)	0.0041 (10)
C45	0.0421 (13)	0.0482 (14)	0.0265 (11)	−0.0076 (11)	0.0026 (9)	−0.0026 (10)
S1	0.0375 (3)	0.0351 (3)	0.0315 (3)	−0.0020 (2)	0.0062 (2)	−0.0083 (2)
C46	0.0346 (11)	0.0272 (10)	0.0259 (10)	−0.0033 (8)	0.0040 (8)	−0.0022 (8)
C47	0.0290 (10)	0.0267 (10)	0.0262 (10)	−0.0021 (8)	0.0016 (8)	−0.0006 (8)
N5	0.0298 (9)	0.0238 (8)	0.0235 (8)	−0.0022 (7)	0.0013 (6)	0.0004 (6)
C48	0.0298 (10)	0.0263 (10)	0.0272 (10)	−0.0021 (8)	0.0025 (8)	−0.0012 (8)
C49	0.0295 (10)	0.0279 (10)	0.0330 (11)	−0.0050 (8)	0.0007 (8)	−0.0005 (8)
C50	0.0353 (11)	0.0290 (10)	0.0274 (10)	−0.0042 (9)	−0.0011 (8)	−0.0036 (8)
S2	0.0295 (3)	0.0330 (3)	0.0409 (3)	−0.0078 (2)	0.0016 (2)	−0.0044 (2)
C51	0.0364 (12)	0.0400 (13)	0.0359 (12)	−0.0034 (10)	−0.0050 (9)	−0.0057 (10)
C52	0.0337 (11)	0.0348 (12)	0.0319 (11)	−0.0006 (9)	−0.0038 (9)	−0.0012 (9)
C53	0.0292 (11)	0.0410 (13)	0.0483 (14)	−0.0059 (10)	0.0012 (10)	−0.0056 (11)
C54	0.0293 (11)	0.0401 (13)	0.0437 (13)	0.0009 (9)	0.0044 (9)	−0.0044 (10)
C55	0.0292 (10)	0.0269 (10)	0.0314 (11)	0.0030 (8)	−0.0021 (8)	0.0034 (8)
C56	0.0316 (10)	0.0309 (11)	0.0298 (10)	−0.0011 (8)	0.0000 (8)	0.0042 (8)
C57	0.0360 (11)	0.0348 (12)	0.0275 (10)	−0.0019 (9)	0.0013 (8)	0.0008 (9)
C58	0.0292 (10)	0.0248 (10)	0.0306 (10)	0.0023 (8)	0.0042 (8)	0.0034 (8)
C59	0.0282 (10)	0.0329 (11)	0.0391 (12)	0.0056 (9)	0.0002 (9)	0.0028 (9)
C60	0.0371 (12)	0.0277 (11)	0.0426 (13)	0.0094 (9)	0.0086 (10)	0.0027 (9)
C61	0.0437 (13)	0.0261 (10)	0.0333 (11)	0.0045 (9)	0.0038 (9)	−0.0016 (9)
C62	0.0341 (11)	0.0274 (10)	0.0304 (11)	0.0034 (8)	0.0002 (8)	0.0009 (8)
C63	0.0271 (9)	0.0234 (9)	0.0259 (10)	0.0016 (7)	0.0044 (7)	0.0029 (7)
C71	0.067 (4)	0.053 (3)	0.063 (4)	−0.004 (3)	0.004 (3)	0.005 (3)
Cl1	0.0902 (15)	0.0825 (15)	0.0762 (13)	−0.0186 (13)	−0.0003 (11)	0.0025 (12)
Cl2	0.0951 (16)	0.0745 (14)	0.0752 (13)	0.0037 (12)	−0.0048 (11)	−0.0074 (11)

### Geometric parameters (Å, º)

**Table:**

Ni1—N4	2.0350 (17)	C34—H34	0.9500
Ni1—N3	2.0402 (17)	C35—C36	1.388 (4)
Ni1—N1	2.0407 (17)	C35—H35	0.9500
Ni1—N2	2.0434 (17)	C36—C37	1.381 (4)
Ni1—N5	2.1122 (17)	C36—H36	0.9500
N1—C4	1.365 (3)	C37—C38	1.395 (3)
N1—C1	1.372 (3)	C37—H37	0.9500
N2—C9	1.372 (3)	C38—C39	1.487 (3)
N2—C6	1.372 (3)	C39—C44	1.392 (3)
N3—C11	1.370 (3)	C39—C40	1.395 (4)
N3—C14	1.371 (3)	C40—C41	1.380 (4)
N4—C19	1.370 (3)	C40—H40	0.9500
N4—C16	1.374 (3)	C41—C42	1.393 (4)
C1—C20	1.393 (3)	C41—H41	0.9500
C1—C2	1.444 (3)	C42—C43	1.388 (4)
C2—C3	1.341 (3)	C42—C45	1.496 (3)
C2—H2	0.9500	C43—C44	1.385 (4)
C3—C4	1.446 (3)	C43—H43	0.9500
C3—H3	0.9500	C44—H44	0.9500
C4—C5	1.398 (3)	C45—S1	1.839 (3)
C5—C6	1.398 (3)	C45—H45A	0.9900
C5—C21	1.495 (3)	C45—H45B	0.9900
C6—C7	1.440 (3)	S1—C46	1.776 (2)
C7—C8	1.349 (3)	C46—C50	1.388 (3)
C7—H7	0.9500	C46—C47	1.395 (3)
C8—C9	1.442 (3)	C47—N5	1.347 (3)
C8—H8	0.9500	C47—H47	0.9500
C9—C10	1.399 (3)	N5—C48	1.343 (3)
C10—C11	1.399 (3)	C48—C49	1.394 (3)
C10—C63	1.498 (3)	C48—H48	0.9500
C11—C12	1.439 (3)	C49—C50	1.387 (3)
C12—C13	1.351 (3)	C49—S2	1.770 (2)
C12—H12	0.9500	C50—H50	0.9500
C13—C14	1.439 (3)	S2—C51	1.837 (3)
C13—H13	0.9500	C51—C52	1.492 (3)
C14—C15	1.395 (3)	C51—H51A	0.9900
C15—C16	1.395 (3)	C51—H51B	0.9900
C15—C33	1.501 (3)	C52—C53	1.390 (3)
C16—C17	1.443 (3)	C52—C57	1.396 (3)
C17—C18	1.348 (3)	C53—C54	1.386 (4)
C17—H17	0.9500	C53—H53	0.9500
C18—C19	1.442 (3)	C54—C55	1.394 (3)
C18—H18	0.9500	C54—H54	0.9500
C19—C20	1.397 (3)	C55—C56	1.395 (3)
C20—C27	1.497 (3)	C55—C58	1.481 (3)
C21—C22	1.382 (4)	C56—C57	1.388 (3)
C21—C26	1.389 (3)	C56—H56	0.9500
C22—F1	1.338 (3)	C57—H57	0.9500
C22—C23	1.387 (4)	C58—C59	1.399 (3)
C23—F2	1.336 (4)	C58—C63	1.401 (3)
C23—C24	1.372 (5)	C59—C60	1.383 (3)
C24—F3	1.344 (3)	C59—H59	0.9500
C24—C25	1.380 (5)	C60—C61	1.383 (4)
C25—F4	1.339 (3)	C60—H60	0.9500
C25—C26	1.382 (3)	C61—C62	1.390 (3)
C26—F5	1.335 (3)	C61—H61	0.9500
C27—C28	1.381 (3)	C62—C63	1.398 (3)
C27—C32	1.388 (3)	C62—H62	0.9500
C28—F6	1.341 (3)	C71—C71^i^	1.126 (13)
C28—C29	1.382 (4)	C71—Cl1^i^	1.326 (8)
C29—F7	1.331 (3)	C71—Cl2^i^	1.607 (8)
C29—C30	1.381 (5)	C71—Cl1	1.753 (7)
C30—F8	1.338 (3)	C71—Cl2	1.772 (8)
C30—C31	1.368 (5)	C71—H71A	0.9600
C31—F9	1.343 (3)	C71—H71B	0.9599
C31—C32	1.380 (3)	Cl1—Cl2^i^	0.882 (3)
C32—F10	1.339 (3)	Cl1—C71^i^	1.326 (8)
C33—C34	1.391 (3)	Cl2—Cl1^i^	0.882 (3)
C33—C38	1.402 (3)	Cl2—C71^i^	1.607 (8)
C34—C35	1.383 (3)		
			
N4—Ni1—N3	89.66 (7)	C34—C35—H35	120.2
N4—Ni1—N1	89.03 (7)	C36—C35—H35	120.2
N3—Ni1—N1	166.05 (7)	C37—C36—C35	119.8 (2)
N4—Ni1—N2	165.76 (7)	C37—C36—H36	120.1
N3—Ni1—N2	88.58 (7)	C35—C36—H36	120.1
N1—Ni1—N2	89.29 (7)	C36—C37—C38	121.2 (2)
N4—Ni1—N5	96.84 (7)	C36—C37—H37	119.4
N3—Ni1—N5	100.53 (7)	C38—C37—H37	119.4
N1—Ni1—N5	93.42 (7)	C37—C38—C33	118.8 (2)
N2—Ni1—N5	97.37 (7)	C37—C38—C39	121.1 (2)
C4—N1—C1	106.09 (17)	C33—C38—C39	120.09 (19)
C4—N1—Ni1	126.63 (14)	C44—C39—C40	118.1 (2)
C1—N1—Ni1	126.60 (14)	C44—C39—C38	121.5 (2)
C9—N2—C6	105.94 (16)	C40—C39—C38	120.4 (2)
C9—N2—Ni1	127.28 (13)	C41—C40—C39	121.0 (2)
C6—N2—Ni1	126.72 (14)	C41—C40—H40	119.5
C11—N3—C14	105.91 (16)	C39—C40—H40	119.5
C11—N3—Ni1	127.38 (13)	C40—C41—C42	120.6 (3)
C14—N3—Ni1	126.60 (14)	C40—C41—H41	119.7
C19—N4—C16	106.08 (16)	C42—C41—H41	119.7
C19—N4—Ni1	127.29 (14)	C43—C42—C41	118.5 (2)
C16—N4—Ni1	126.52 (14)	C43—C42—C45	120.9 (2)
N1—C1—C20	125.30 (19)	C41—C42—C45	120.4 (2)
N1—C1—C2	109.85 (18)	C44—C43—C42	120.8 (2)
C20—C1—C2	124.85 (19)	C44—C43—H43	119.6
C3—C2—C1	107.04 (19)	C42—C43—H43	119.6
C3—C2—H2	126.5	C43—C44—C39	120.8 (2)
C1—C2—H2	126.5	C43—C44—H44	119.6
C2—C3—C4	107.00 (19)	C39—C44—H44	119.6
C2—C3—H3	126.5	C42—C45—S1	112.03 (17)
C4—C3—H3	126.5	C42—C45—H45A	109.2
N1—C4—C5	125.35 (19)	S1—C45—H45A	109.2
N1—C4—C3	110.00 (18)	C42—C45—H45B	109.2
C5—C4—C3	124.64 (19)	S1—C45—H45B	109.2
C4—C5—C6	125.46 (19)	H45A—C45—H45B	107.9
C4—C5—C21	117.01 (18)	C46—S1—C45	101.61 (11)
C6—C5—C21	117.52 (19)	C50—C46—C47	118.6 (2)
N2—C6—C5	125.22 (18)	C50—C46—S1	120.23 (17)
N2—C6—C7	110.09 (18)	C47—C46—S1	120.85 (17)
C5—C6—C7	124.68 (19)	N5—C47—C46	122.5 (2)
C8—C7—C6	106.97 (19)	N5—C47—H47	118.7
C8—C7—H7	126.5	C46—C47—H47	118.7
C6—C7—H7	126.5	C48—N5—C47	118.15 (18)
C7—C8—C9	106.97 (19)	C48—N5—Ni1	120.05 (14)
C7—C8—H8	126.5	C47—N5—Ni1	121.32 (14)
C9—C8—H8	126.5	N5—C48—C49	123.0 (2)
N2—C9—C10	125.87 (18)	N5—C48—H48	118.5
N2—C9—C8	110.03 (17)	C49—C48—H48	118.5
C10—C9—C8	124.10 (19)	C50—C49—C48	118.3 (2)
C11—C10—C9	124.16 (18)	C50—C49—S2	120.91 (17)
C11—C10—C63	118.77 (18)	C48—C49—S2	120.56 (17)
C9—C10—C63	117.00 (18)	C49—C50—C46	119.5 (2)
N3—C11—C10	125.76 (18)	C49—C50—H50	120.3
N3—C11—C12	110.18 (18)	C46—C50—H50	120.3
C10—C11—C12	124.03 (19)	C49—S2—C51	101.32 (11)
C13—C12—C11	106.82 (19)	C52—C51—S2	111.40 (17)
C13—C12—H12	126.6	C52—C51—H51A	109.3
C11—C12—H12	126.6	S2—C51—H51A	109.3
C12—C13—C14	106.96 (19)	C52—C51—H51B	109.3
C12—C13—H13	126.5	S2—C51—H51B	109.3
C14—C13—H13	126.5	H51A—C51—H51B	108.0
N3—C14—C15	125.62 (19)	C53—C52—C57	118.3 (2)
N3—C14—C13	110.08 (18)	C53—C52—C51	121.0 (2)
C15—C14—C13	124.29 (19)	C57—C52—C51	120.5 (2)
C16—C15—C14	125.19 (19)	C54—C53—C52	121.1 (2)
C16—C15—C33	116.82 (18)	C54—C53—H53	119.4
C14—C15—C33	117.93 (18)	C52—C53—H53	119.4
N4—C16—C15	125.76 (18)	C53—C54—C55	120.6 (2)
N4—C16—C17	109.77 (18)	C53—C54—H54	119.7
C15—C16—C17	124.46 (19)	C55—C54—H54	119.7
C18—C17—C16	107.14 (19)	C54—C55—C56	118.4 (2)
C18—C17—H17	126.4	C54—C55—C58	121.1 (2)
C16—C17—H17	126.4	C56—C55—C58	120.4 (2)
C17—C18—C19	106.90 (19)	C57—C56—C55	120.8 (2)
C17—C18—H18	126.6	C57—C56—H56	119.6
C19—C18—H18	126.6	C55—C56—H56	119.6
N4—C19—C20	124.95 (19)	C56—C57—C52	120.6 (2)
N4—C19—C18	110.11 (18)	C56—C57—H57	119.7
C20—C19—C18	124.92 (19)	C52—C57—H57	119.7
C1—C20—C19	125.50 (19)	C59—C58—C63	119.0 (2)
C1—C20—C27	116.76 (18)	C59—C58—C55	120.4 (2)
C19—C20—C27	117.69 (18)	C63—C58—C55	120.58 (19)
C22—C21—C26	116.7 (2)	C60—C59—C58	121.2 (2)
C22—C21—C5	121.9 (2)	C60—C59—H59	119.4
C26—C21—C5	121.3 (2)	C58—C59—H59	119.4
F1—C22—C21	120.0 (2)	C61—C60—C59	120.0 (2)
F1—C22—C23	117.8 (3)	C61—C60—H60	120.0
C21—C22—C23	122.2 (3)	C59—C60—H60	120.0
F2—C23—C24	120.5 (3)	C60—C61—C62	119.6 (2)
F2—C23—C22	120.1 (3)	C60—C61—H61	120.2
C24—C23—C22	119.4 (3)	C62—C61—H61	120.2
F3—C24—C23	120.0 (3)	C61—C62—C63	121.0 (2)
F3—C24—C25	119.7 (3)	C61—C62—H62	119.5
C23—C24—C25	120.3 (2)	C63—C62—H62	119.5
F4—C25—C24	120.5 (2)	C62—C63—C58	119.17 (19)
F4—C25—C26	120.3 (3)	C62—C63—C10	119.72 (19)
C24—C25—C26	119.2 (3)	C58—C63—C10	121.01 (19)
F5—C26—C25	117.9 (2)	C71^i^—C71—Cl1^i^	90.9 (8)
F5—C26—C21	119.9 (2)	C71^i^—C71—Cl2^i^	78.7 (7)
C25—C26—C21	122.2 (2)	Cl1^i^—C71—Cl2^i^	167.9 (6)
C28—C27—C32	116.2 (2)	C71^i^—C71—Cl1	49.1 (6)
C28—C27—C20	121.0 (2)	Cl1^i^—C71—Cl1	140.1 (5)
C32—C27—C20	122.8 (2)	Cl2^i^—C71—Cl1	30.03 (17)
F6—C28—C27	119.4 (2)	C71^i^—C71—Cl2	62.8 (7)
F6—C28—C29	117.7 (2)	Cl1^i^—C71—Cl2	28.7 (2)
C27—C28—C29	122.9 (3)	Cl2^i^—C71—Cl2	141.5 (4)
F7—C29—C30	120.3 (3)	Cl1—C71—Cl2	111.7 (4)
F7—C29—C28	120.7 (3)	C71^i^—C71—H71A	121.1
C30—C29—C28	119.1 (3)	Cl1^i^—C71—H71A	90.2
F8—C30—C31	120.2 (3)	Cl2^i^—C71—H71A	90.0
F8—C30—C29	120.0 (3)	Cl1—C71—H71A	109.2
C31—C30—C29	119.7 (2)	Cl2—C71—H71A	109.2
F9—C31—C30	119.4 (2)	C71^i^—C71—H71B	130.2
F9—C31—C32	120.5 (3)	Cl1^i^—C71—H71B	96.4
C30—C31—C32	120.1 (3)	Cl2^i^—C71—H71B	95.1
F10—C32—C31	118.0 (2)	Cl1—C71—H71B	109.3
F10—C32—C27	120.0 (2)	Cl2—C71—H71B	109.3
C31—C32—C27	122.0 (2)	H71A—C71—H71B	108.1
C34—C33—C38	119.5 (2)	Cl2^i^—Cl1—C71^i^	104.9 (4)
C34—C33—C15	119.0 (2)	Cl2^i^—Cl1—C71	65.8 (3)
C38—C33—C15	121.5 (2)	C71^i^—Cl1—C71	39.9 (5)
C35—C34—C33	121.1 (2)	Cl1^i^—Cl2—C71^i^	84.2 (4)
C35—C34—H34	119.5	Cl1^i^—Cl2—C71	46.3 (3)
C33—C34—H34	119.5	C71^i^—Cl2—C71	38.5 (4)
C34—C35—C36	119.6 (2)		

Symmetry code: (i) −*x*+1, −*y*+1, −*z*+1.

### Hydrogen-bond geometry (Å, º)

**Table:**

*D*—H···*A*	*D*—H	H···*A*	*D*···*A*	*D*—H···*A*
C2—H2···S2^i^	0.95	3.02	3.886 (2)	153
C71—H71*B*···N1^i^	0.96	2.61	3.555 (8)	169

Symmetry code: (i) −*x*+1, −*y*+1, −*z*+1.
