# Supplementary figures and images for: Crystal structure of (15,20-bis­(2,3,4,5,6-penta­fluoro­phen­yl)-5,10-{(pyridine-3,5-di­yl)bis­[(sulfane­diyl­methyl­ene)[1,1′-biphen­yl]-4′,2-di­yl]}porph­yrin­ato)nickel(II) di­chloro­methane x-solvate (x > 1/2) showing a rare CN5 coordination
Source: Acta Crystallogr E Crystallogr Commun. 2019 Jul 12;75(Pt 8):1180–4. doi: 10.1107/S2056989019009836 (PMC6690462; doi:10.1107/S2056989019009836)

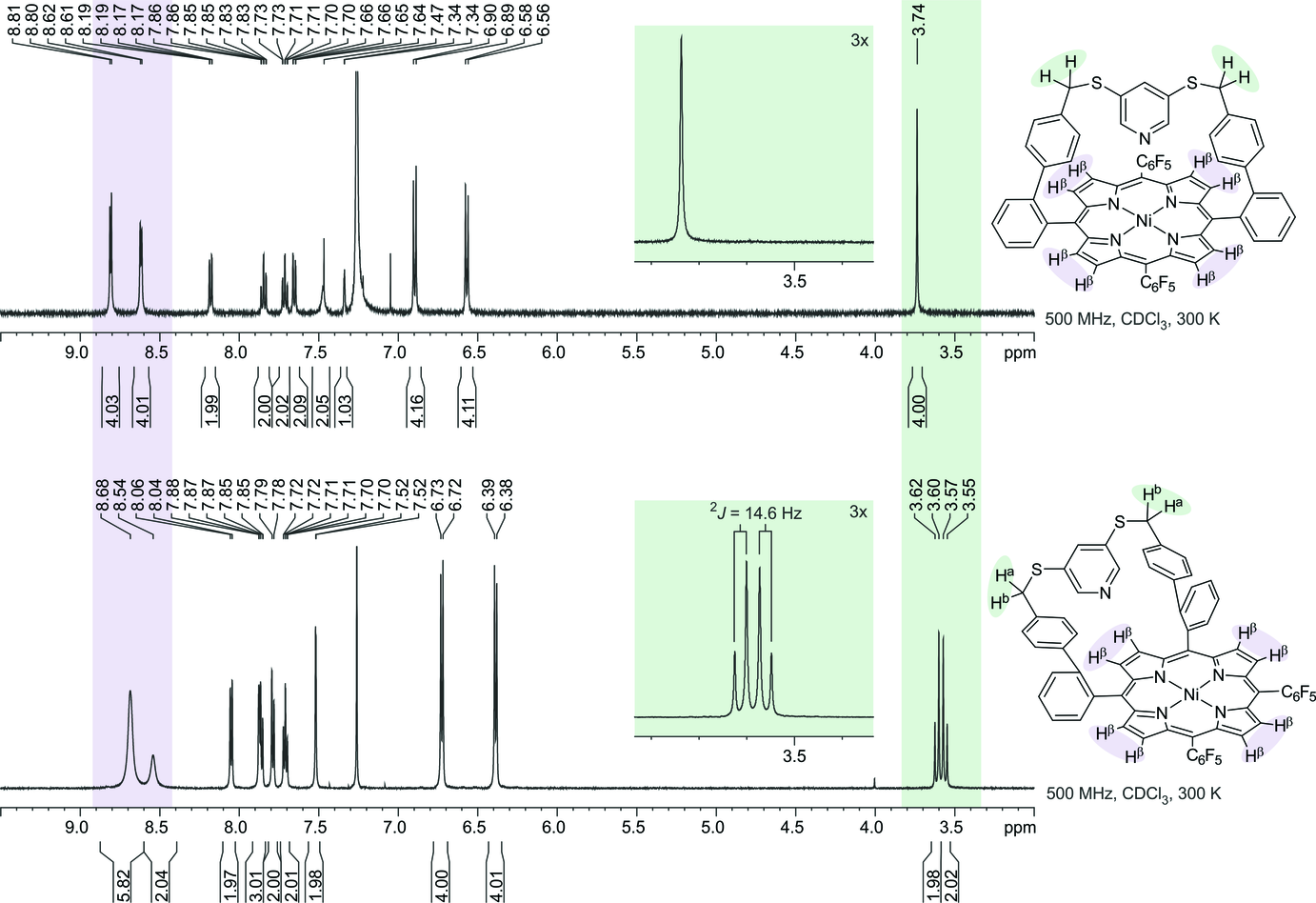

Supplement: Supplementary file 3 [file e-75-01180-sup3.tif]
